# Supplementary material for: Cuticular Hydrocarbon Recognition in the Mating Behavior of Two Pissodes Species
Source: Insects. 2019 Jul 23;10(7):217. doi: 10.3390/insects10070217 (PMC6681335; doi:10.3390/insects10070217)
Supplement: Supplementary file 1 [file insects-10-00217-s001.pdf]

Table S1. The average (+SD) concentration of each CHC component per weevil (μg) of both sexes from the spring and summer populations of both species.

Peak No.	Compound name	LI	LI lib	*P. strobi*				*P. nemorensis*			
				Spring		Summer		Spring		Summer	
				Male	Female	Male	Female	Male	Female	Male	Female
**1**	Pentacosane	2572	2574	2.45 ± 0.36	3.69 ± 0.86	1.29 ± 0.53	0.69 ± 0.16	1.78 ± 0.72	2.79 ± 0.58	2.50 ±1.29	3.47 ± 1.62
**2**	3-Methylpentacosane			0.12 ± 0.07	0.40 ± 0.05	0.05 ± 0.03	1.49 ± 0.94	ND	0.05 ± 0.05	ND	0.37 ± 0.24
**3**	Hexacosane			0.68 ± 0.06	1.00 ± 0.21	1.41 ± 0.54	3.10 ± 1.37	0.60 ± 0.49	0.77 ± 0.23	2.42 ± 0.93	1.91 ± 0.35
**4**	6,9-Heptacosadiene	2674	2676	0.07 ± 0.07	0.21 ± 0.16	0.09 ± 0.06	ND	0.04 ± 0.04	0.32 ± 0.22	0.12 ± 0.07	0.16 ± 0.11
**5**	Heptacosane			18.02 ± 1.56	19.17 ± 4.45	10.23 ± 1.72	5.96 ± 1.02	15.95 ± 4.15	19.43 ± 3.67	7.76 ± 0.37	5.30 ± 0.88
**6**	13-Methylheptacosane	2730	2733	2.63 ± 0.61	2.17 ± 0.60	1.21 ± 0.26	1.91 ± 0.76	1.10 ± 0.66	1.94 ± 0.66	1.89 ± 0.56	1.09 ± 0.36
**7**	11,15-Dimethylheptacosane	2757	2757	0.77 ± 0.26	0.83 ± 0.22	ND	ND	0.12 ± 0.12	0.39 ± 0.32	ND	ND
**8**	3-Methylheptacosane	2772	2773	3.29 ± 0.83	3.95 ± 0.28	1.36 ± 0.35	0.84 ± 0.32	1.82 ± 0.78	3.61 ± 0.37	0.68 ± 0.22	1.11 ± 0.36
**9**	Octacosane			2.44 ± 0.22	2.57 ± 0.34	5.92 ± 0.74	3.26 ± 0.51	2.66 ± 0.48	2.86 ± 0.30	5.21 ± 0.67	3.42 ± 0.57
**10**	6,9-Nonacosadiene	2876	2876	4.31 ± 0.89	8.06 ± 1.45	6.36 ± 1.63	5.54 ± 0.87	3.09 ± 0.61	5.52 ± 0.55	5.11 ± 1.15	7.90 ± 1.14
**11**	Nonacosane			10.67 ± 1.29	6.03 ± 0.68	21.35 ± 4.50	12.74 ± 3.60	20.41 ± 6.99	6.97 ± 1.37	18.43 ± 1.79	9.72 ± 0.74
**12**	13-Methylnonacosane	2930	2931	4.76 ± 0.12	4.55 ± 0.52	1.94 ± 0.46	1.90 ± 0.45	4.00 ± 0.79	5.19 ± 0.50	3.01 ± 0.61	2.67 ± 0.43
**13**	5-Methylnonacosane	2956	2951	0.31 ± 0.10	0.82 ± 0.22	ND	ND	ND	0.31 ± 0.20	ND	ND
**14**	3-Methylnonacosane	2973	2973	2.32 ± 0.53	2.92 ± 0.37	4.24 ± 0.47	3.17 ± 0.66	2.36 ± 1.38	2.20 ± 0.67	3.36 ± 0.30	3.48 ± 0.92
**15**	10-Hentriacontane	3077	3081	6.72 ± 1.10	7.23 ± 0.70	7.83 ± 1.38	8.25 ± 0.56	5.56 ± 0.62	5.12 ± 0.23	7.71 ± 0.84	6.71 ± 1.69
**16**	13-Methylhentriacontane	3127	3129	7.24 ± 1.70	4.90 ± 0.44	7.95 ± 3.92	12.92 ± 3.02	5.76 ± 1.22	5.89 ± 0.55	4.67 ± 0.41	5.53 ± 0.37
**17**	5-Methylhentriacontane	3151	3150	7.07 ± 1.21	6.39 ± 0.58	6.76 ± 1.07	8.60 ± 0.32	6.36 ± 1.37	9.58 ± 1.54	6.42 ± 0.20	9.80 ± 2.08
**18**	Tritriacontadiene	3252	3256	0.74 ± 0.09	0.67 ± 0.12	1.54 ± 0.38	2.55 ± 0.51	0.52 ± 0.20	0.92 ± 0.31	1.37 ± 0.08	1.57 ± 1.61
**19**	9-Tritriacotadiene	3278	3277	4.16 ± 1.43	2.48 ± 0.18	3.36 ± 0.46	3.86 ± 0.51	2.93 ± 0.62	2.22 ± 0.39	3.52 ± 0.28	4.08 ± 0.49
**20**	15-Methyltritriacontane	3327	3327	2.62 ± 0.29	2.18 ± 0.43	2.01 ± 0.48	2.67 ± 0.18	2.21 ± 0.41	2.77 ± 0.29	2.96 ± 0.51	3.66 ± 0.28
**21**	5-Methyltritriacontane	3352	3350	18.61 ± 1.62	19.78 ± 3.29	15.08 ± 2.62	20.55 ± 0.64	22.68 ± 4.76	21.13 ± 2.86	22.87 ± 2.28	28.03 ± 3.02

Mean± SD; n = 5; "LI" represents the calculated linear retention indices in the study, "LI lib" represent the reference found in NIST library.
